# Syndecan-1-Dependent Regulation of Heparanase Affects Invasiveness, Stem Cell Properties, and Therapeutic Resistance of Caco2 Colon Cancer Cells

**DOI:** 10.3389/fonc.2020.00774

**Published:** 2020-05-14

**Authors:** Sampath Kumar Katakam, Paride Pelucchi, Cinzia Cocola, Rolland Reinbold, Israel Vlodavsky, Burkhard Greve, Martin Götte

**Affiliations:** ^1^Department of Gynecology and Obstetrics, Münster University Hospital, Münster, Germany; ^2^Institute of Biomedical Technologies, National Research Council, Milan, Italy; ^3^The Rappaport Faculty of Medicine, Technion Integrated Cancer Center (TICC), Haifa, Israel; ^4^Department of Radiotherapy-Radiooncology, Münster University Hospital, Münster, Germany

**Keywords:** heparanase, syndecan, colon cancer, cancer stem cells, heparan sulfate, proteoglycan

## Abstract

The heparan sulfate proteoglycan Syndecan-1 binds cytokines, morphogens and extracellular matrix components, regulating cancer stem cell properties and invasiveness. Syndecan-1 is modulated by the heparan sulfate-degrading enzyme heparanase, but the underlying regulatory mechanisms are only poorly understood. In colon cancer pathogenesis, complex changes occur in the expression pattern of Syndecan-1 and heparanase during progression from well-differentiated to undifferentiated tumors. Loss of Syndecan-1 and increased expression of heparanase are associated with a change in phenotypic plasticity and an increase in invasiveness, metastasis and dedifferentiation. Here we investigated the regulatory and functional interplay of Syndecan-1 and heparanase employing siRNA-mediated silencing and plasmid-based overexpression approaches in the human colon cancer cell line Caco2. Heparanase expression and activity were upregulated in Syndecan-1 depleted cells. This increase was linked to an upregulation of the transcription factor Egr1, which regulates heparanase at the promoter level. Inhibitor experiments demonstrated an impact of focal adhesion kinase, Wnt and ROCK-dependent signaling on this process. siRNA-depletion of Syndecan-1, and upregulation of heparanase increased the colon cancer stem cell phenotype based on sphere formation assays and phenotypic marker analysis (Side-population, NANOG, KLF4, NOTCH, Wnt, and TCF4 expression). Syndecan-1 depletion increased invasiveness of Caco2 cells *in vitro* in a heparanase-dependent manner. Finally, upregulated expression of heparanase resulted in increased resistance to radiotherapy, whereas high expression of enzymatically inactive heparanase promoted chemoresistance to paclitaxel and cisplatin. Our findings provide a new avenue to target a stemness-associated signaling axis as a therapeutic strategy to reduce metastatic spread and cancer recurrence.

## Introduction

In the tumor microenvironment (TME), repopulation of cells after radiotherapy and chemotherapy represents a mechanism of resistance and tumor recurrence (1). Abnormal changes in extracellular matrix (ECM) components and their degradative enzymes causes an imbalance between tissue homeostasis and cancer, resulting in changes in cell plasticity associated with increased invasion, metastasis and dedifferentiation (2). The “metastatic niche” is regulated by the “cancer stem cell niche” with abnormal changes in ECM dynamics (2–4). For example, heparanase (HPSE), matrix metalloproteinases, and sulfatases, are highly expressed in many cancers, whereas some heparan sulfate sulfotransferases are silenced (5–7). Indeed, proteoglycan-degrading enzymes such as HPSE, the only mammalian endoglycosidase capable of cleaving heparan sulfate, regulate ECM dynamics that are under the tight homeostatic control of several signaling pathways (7, 8). Recent studies indicate that the interplay between the cell surface proteoglycan Syndecan-1 (Sdc-1) and HPSE have important functional connections in the progression of colorectal cancer and myeloma. For example, in colon cancer progression, there is a gradual increase in the expression of HPSE (9) and a decrease in Sdc-1 (10) expression during progression from well-differentiated to poorly differentiated colon carcinoma. Differences in the mRNA and protein expression of Sdc-1 have been noted, as Sdc-1 mRNA was strongly overexpressed in metastatic colon tumors, whereas using immunohistochemistry, metastatic tumors showed a dramatic decrease in staining, while labeling was still strong in the adjacent normal mucosa (11, 12). Moreover, in metastatic tumors HPSE mRNA levels were reduced in 40% of patients, whereas overexpression was observed in 20% of patients, indicating considerable heterogeneity (11). Deeply invading colon carcinoma cells showed decreased expression of Sdc-1 (13) and increased expression of HPSE (14, 15). Consistent with these findings, the malignant transformation of Caco2 colon carcinoma cells resulted in a decrease in the Sdc-1 expression (15) which might also regulate HPSE activity. Transcriptional studies show that loss of Sdc-1 (13, 16) and enhanced expression of HPSE (17–19) correlate with tumor growth, invasion, metastatic potential, and reduced postoperative survival of cancer patients (20). In colitis and the associated tumorigenic models, the transcriptional regulator early growth response 1 (EGR1) acts as a potent inducer of HPSE in colonic epithelial tumor cells (17, 21, 22). While Sdc-1 expression maintains epithelial integrity, loss of expression results in high HPSE expression, changes in epithelial morphology and polarity, thereby promoting epithelial-mesenchymal transition (EMT) (23). Thus, Sdc-1 and HPSE work together to enhance cell invasiveness via EMT pathways, which may further enhance stem cell-like pluripotency signatures (24, 25). As EMT regulates metastasis (26–28), high expression of HPSE may further enhance metastasis based on the concept of migrating cancer stem cells (CSCs). Data in different tumor entities have revealed further pathogenetic mechanisms for the functional interplay of Sdc-1 and HPSE. For example, in multiple myeloma, high HPSE expression is linked to poor prognosis, and contributes to disease pathogenesis by inducing Sdc-1 shedding from the tumor cell membrane (29), which promotes sequestering of shed Sdc-1 bound growth factors in the tumor microenvironment (30). Additional molecular mechanisms linked to HPSE overexpression include activation of the Erk signaling pathway, the reduction of nuclear Sdc-1 leading to increased acetylated histone H3 and subsequent upregulation of vascular endothelial growth factor (VEGF) and matrix metalloproteinase (MMP)-9 (30). Finally, both HPSE and Sdc-1 regulate the activity of pathways relevant to cancer progression, such as the stemness-associated Wnt pathway (3, 31, 32) and metastasis-related focal adhesion kinase (FAK) signaling (3, 33, 34).

Although high HPSE expression in various solid tumors confers resistance to stress and chemo/radiotherapy (35–37), its role in promoting tumor initiation via the expression of CSC-like signatures has not been elucidated. Owing to the role of Sdc-1 and HPSE in tumor growth, invasion and metastasis we aimed at investigating the underlying molecular interplay between Sdc-1 and HPSE and the possible signaling routes in the well-established colon cancer cell line Caco2, applying both stable overexpression and transient siRNA knockdown methods. Our results report for the first time the dynamic interplay between Sdc-1 and HPSE in stemness-associated colon cancer via a signaling axis involving early growth response protein 1 (EGR1), FAK, and Wnt. Our findings could form a conceptual framework for establishing novel therapeutic possibilities and recognize the long-term driven functions of Sdc-1 and HPSE in colon cancer.

## Materials and Methods

### Materials

Tissue culture supplies were from Gibco BRL (Karlsruhe, Germany). Unless stated otherwise, all chemicals were from Sigma Aldrich (Deisenhofen, Germany).

### Cell Culture

The human colon carcinoma cell line Caco2 (German Collection of Microorganisms and Cell Cultures, Department of Human and Animal Cell Cultures, Braunschweig, Germany) was stably transfected with a pcDNA3.1 control plasmid (Invitrogen), or plasmids overexpressing Syndecan-1 cDNA (38), native HPSE, or enzymatically inactive HPSE double mutated in Glu225 and Glu343 (39). Stable clones were selected using 800 μg/ml G418. Caco2 cells were maintained in RPMI media containing 10% fetal calf serum (FCS), 1% glutamine, 1% penicillin/streptomycin and 800 μg/ml G418 in a humidified atmosphere of 5% CO_2_ at 37°C. Successful transfection was confirmed by qPCR.

### siRNA Knockdown of Syndecan-1 and Egr1 Expression

siRNA knockdown was performed using pre-validated siRNAs #12634 and # 4537 (Ambion, Cambridgeshire, UK) targeting the coding regions of Syndecan-1, and EGR1, respectively, and a negative control siRNA (negative control #1, Ambion). In preliminary experiments, we optimized conditions for the efficient transfection of Caco2 cells. Fresh medium was added 16 h after transfection, and experiments were conducted 48 h after transfection. Target downregulation was confirmed by qPCR.

### Cell viability and Chemosensitivity Assay

Cell viability was evaluated by MTT (3-(4,5-dimethylthiazol-2-yl)-2,5-diphenyltetrazolium bromide) assay exactly as previously described (6). To test chemosensitivity, the MTT assay was performed in the presence of titrated concentrations of Paclitaxel (10 pM−1 μM), and cis-diamineplatinum II dichloride (50 nM−5 mM), which were added 24 h after initial cell plating.

### Invasion Assay

BioCoat Matrigel Invasion Chamber (BD Biosciences, Heidelberg, Germany) assays are based on the chemotaxis-driven invasion of porous filter membranes coated with a basement membrane-like matrix. Assays were performed exactly as previously described (6) using an invasion time of 4 days. For inhibitor studies, SST0001 (1 μg/ml; = Roneparstat) (40, 41) was added to both compartments 24 h after cell plating. Relative invasiveness was expressed as percentage of the cell number on compound-treated inserts compared with control inserts. The invasion experiments were performed and analyzed by two different researchers (SKK, BP).

### Quantitative Real-Time PCR

Total cellular RNA was isolated using rna-OLS (OMNI Life Science, Hamburg, Germany) and reverse transcribed (Advantage First strand cDNA synthesis kit; Fermentas, St. Leon-Rot, Germany). qPCR and melting curve analysis were performed using Qiagen QuantiTect SYBR Green PCR kit in a LightCycler (Roche, IN). Expression of additional mRNAs was analyzed using the following TaqMan probes on an ABI PRISM 7300 Sequence Detection System, as described previously (42): 18S rRNA Hs99999901_s1, KLF4 Hs00358836_m1, SDC1 Hs00174579_m1, HPSE Hs00180737_m1. The 2^−ΔΔ*Ct*^ method was used to determine relative gene transcript levels after normalization to 18S rRNA. PF-562271 (Sigma-Aldrich) was used for 24 h at 10 μg/ml in some experiments.

### Western Blot and Immunoprecipitation

Immunoblotting was performed exactly as previously described (6, 42), using the following primary antibodies (1:1,000): rabbit polyclonal anti-phospho FAK Y925 (Cell Signaling, Beverly, MA, USA), rabbit polyclonal anti-FAK (Cell Signaling), rabbit monoclonal anti-human TCF4 (Cell Signaling), mouse anti-E-cadherin (1:2,000; BD Biosciences), mouse anti-human α-Tubulin (Sigma-Aldrich) and appropriate secondary antibodies (diluted 1:5,000): HRP-conjugated goat-anti-mouse or goat-anti-rabbit IgG (Merck-Millipore, Darmstadt, Germany). For immunoprecipitation, cell lysates of Caco2 cells were prepared 72 h after transfection with control or Sdc-1 siRNA as described previously (42). 0.5 mg protein was incubated with 1:50 dilution of primary antibody (rabbit monoclonal anti-human EGR1, Cell Signaling) at 4°C on a rocker platform overnight. Afterward, the mixture was incubated analogously with 20 μl resuspended protein A/G-PLUS-Agarose. Immunoprecipitates were pelleted by centrifugation (1,000 g, 5 min, 4°C), washed four times with RIPA buffer and boiled in 40 μl SDS sample buffer (5 min). SDS-PAGE, Western blotting, stripping and reprobing were performed as described previously (6) using 30–60 μg of protein/lane on 7.5– 12% gels.

### Side Population Analysis

Side population (SP) analysis was performed using the Hoechst 33342 dye exclusion technique as previously described (43). In this assay, a putative CSC population is identified based on the dye efflux properties of ATP-binding cassette (ABC) transporters, which are highly expressed in these cells (44). In some experiments, the inhibitors IWP-2 (10 μM) and SST0001 (10 μg/ml) were used for 1 h prior to SP analysis. 1 × 10^6^ cells were incubated in DMEM containing 2% (v/v) FCS for 90 min at 37°C either with 5 μg/ml Hoechst 33342 (Sigma-Aldrich) or in the presence of 50 μM verapamil (Sigma-Aldrich). Finally, 2 μg/ml propidium iodide was added for cell death discrimination, and cells were stored on ice until analysis. Cells were analyzed on a CyFlow Space (Sysmex/Partec) using a 16 mW 375 nm UV laser for excitation, emission was measured at 475 nm (BP 455/50) and at 665 nm (LP 665 nm). Signals were slivered by a dichroic mirror of 610 nm to measure Hoechst signal intensity in both channels. All cells with a low Hoechst fluorescence and which were not visible in the verapamil control were gated (R2) as SP cells. Data acquisition and processing were done by using FloMax software (Quantum Analysis, Münster, Germany).

### Sphere Culture of Caco2 Cells

Sphere suspension cultures of Caco2 cells were performed in a serum-free medium (RPMI, High Glucose, GlutaMAX™Gibco®), supplemented with B27 (Gibco®), 20 ng/ml EGF (Sigma) and 20 ng/ml basic fibroblast growth factor (bFGF, Immunotools) at a density of 1 x 10^3^ cells/ml. Sphere cultures were performed and analyzed by three independent researchers (PP, CC, RR).

### Irradiation

Irradiation was performed at room temperature with a linear accelerator using a dose rate of 4.8 Gy min^−1^ and a dose of 2 Gy was applied. To measure the colony-forming ability after irradiation, 1 x 10^3^ cells were resuspended in 1 ml culture medium, plated into 3.5 cm Petri dishes with a 2.5 mm grid (Nunc, Langenselbold, Germany) and incubated for about 6 days in a CO_2_ incubator at 37°C. Cell colonies with more than 50 cells were counted using a microscope (Olympus, Hamburg, Germany). The survival fraction was calculated as follows: plating efficiency treated/plating efficiency control. Radiation resistance was analyzed by two independent researchers (SKK, AvD).

### Promoter Reporter Assay

The 1.9-kb human heparanase promoter region [HPSE (-1791/+109)-LUC] was subcloned upstream of the LUC gene in a pGL2 basic reporter plasmid (Promega, Madison, WI, USA) (45, 46). 24 h after siRNA transfection, cells were replaced with serum-free media for 6 h and co-transfected with a reporter construct at 1 μg/well (6 well) using FuGENE 6 reagent (Promega) according to the standard protocol. Control cells were transfected with basic pGL2 plasmid containing LUC gene alone (without promoter). 46 h after transfection, luciferase assay was done using the Luciferase Reporter Assay system. (Promega-E1500). The relative light units were determined in each sample with a luminometer and results were normalized against beta-galactosidase activity measured by a colorimetric assay. Data are presented as the means of quadruplicates ± s.d., and all experiments were repeated at least three times with similar results.

### HPSE Activity Assay

HPSE activity of 1 × 10^6^ cells was measured using a commercial heparan sulfate degrading enzyme assay kit (Takara.Mirus.Bio, Madison, WI) which is based on the measurement of HPSE-induced degradation of biotinylated-HS (b-HS) fragments, according to the manufacturer's protocol.

### Statistical Analysis

Unless indicated otherwise, data were analyzed using the unpaired two-tailed Student's *t*-test. A *P* < 0.05 was considered statistically significant. All experiments were performed at least three times on independent biological replicates.

## Results

### Syndecan-1 Regulates Heparanase Expression and Caco2 Cell Invasiveness

**Figure 1:**
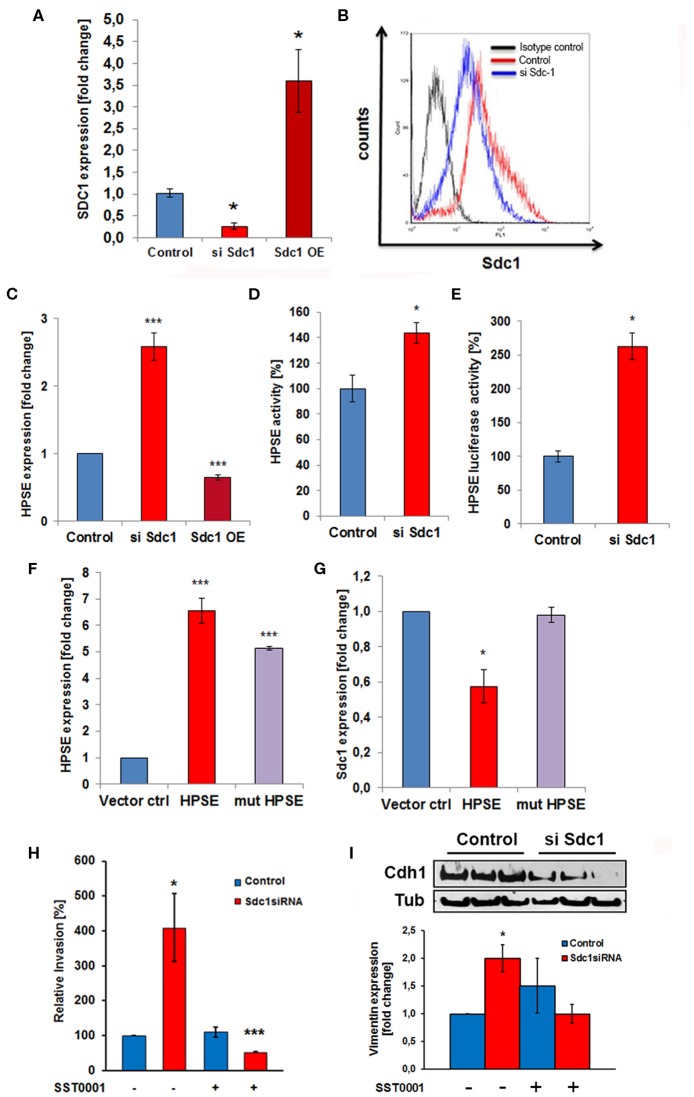
The heparan sulfate proteoglycan Syndecan-1 regulates Caco2 cell invasiveness in a heparanase-dependent manner. **(A)** Confirmation of Sdc-1 siRNA knockdown and overexpression by qPCR. *p < 0.05 vs. all groups. **(B)** Confirmation of Sdc-1 siRNA knockdown by flow cytometry. **(C)** siRNA knockdown (siSdc1) or plasmid-mediated overexpression (OE) of Sdc-1 leads to up-or downregulation of HPSE expression, respectively (qPCR). ***p < 0.001 vs. all groups. **(D,E)** Sdc-1 knockdown results in an upregulation of HPSE enzymatic activity **(D)** and a substantial 3-fold activation of HPSE promoter activity (**E**, luciferase reporter assay). *p < 0.05 vs. control. **(F,G)** Plasmid-mediated overexpression of enzymatically active HPSE (HPSE) results in a decrease of Sdc-1 expression (qPCR). Overexpression of an enzymatically inactive HPSE variant (mut-HPSE) did not affect Sdc-1 expression. ***p < 0.01 vs. control. **(H)** Caco2 cell invasion is stimulated in response to Sdc-1silencing. The HPSE inhibitor SST0001 abolishes the increased Matrigel invasiveness of Sdc-1 siRNA-treated Caco2 cells. *p < 0.05 vs. control. **(I)** Sdc-1 siRNA knockdown affects the expression of the EMT markers E-cadherin and vimentin. Upper panel: Western blotting demonstrates downregulation of the epithelial marker E-cadherin (Cdh1) upon Sdc-1 silencing. Tubulin (Tub) = loading control. Representative picture of three independent experiments. Lower panel: qPCR analysis reveals upregulation of the mesenchymal marker vimentin upon Sdc-1-silencing, which could be reversed by the HPSE inhibitor SST0001. *p < 0.05 vs. untreated control and treated Sdc1 siRNA. All panels *N* ≥ 3. Error bars = SEM.

**Figure 2:**
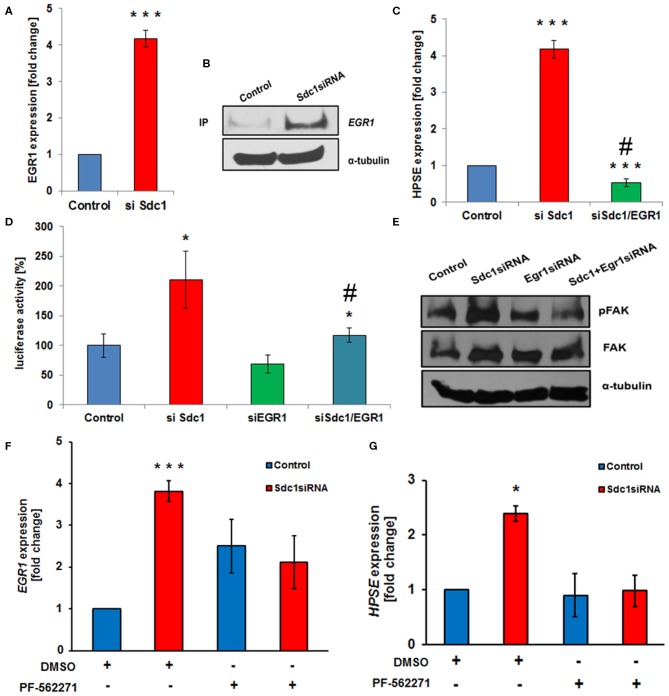
Syndecan-1 regulates HPSE expression in Caco2 cells in an Egr1 and focal adhesion kinase (FAK)-dependent manner. **(A,B)** Sdc1 siRNA knockdown results in a substantial upregulation of the transcriptional regulator Egr1, as demonstrated by qPCR **(A)** and Western blotting **(B)**. ***p < 0.001 vs. control. **(C,D)** Egr1 siRNA knockdown abolishes the Sdc1 siRNA-induced upregulation of HPSE as demonstrated by qPCR **(C)** and HPSE-promoter-based luciferase reporter assays **(D)**. **(C)** ***p < 0.001 vs. control, #*p* < 0.001 vs. si Sdc-1. **(D)** ***p < 0.001 vs. control, #*p* = 0.06 vs. si Sdc-1. **(E)** Egr1 siRNA depletion inhibits the activation of FAK phosphorylation induced by Sdc-1 siRNA knockdown (Western blot). **(F,G)** The FAK inhibitor PF-562271 prevents the Sdc-1-knockdown-induced upregulation of Egr1 **(F)** and HPSE **(G)** (qPCR). **(E)** ***p < 0.001 vs. control, **(F)** *p < 0.05 vs. all groups. All panels: *N* ≥ 3. Error bars = SEM.

Based on the deregulated expression of Sdc-1 and HPSE in colon cancer and the role of Sdc-1 as a signaling co-receptor, we hypothesized that loss of Sdc-1 may regulate HPSE expression. To test our hypothesis, we manipulated Sdc-1 levels via siRNA knockdown in the human colon cancer cell line Caco2. Sdc-1 knockdown (Figures 1A,B) resulted in a substantial increase in HPSE mRNA expression (Figure 1C), HPSE activity (Figure 1D) and HPSE promoter activity (Figure 1E). Consistently, plasmid-based Sdc-1 overexpression was associated with HPSE downregulation (Figures 1A,C). Conversely, plasmid-based overexpression of HPSE induced a reduction of Sdc-1 expression, whereas upregulation of an enzymatically inactive form of HPSE had no effect (Figures 1F,G). At the functional level, Sdc-1 knockdown resulted in increased invasiveness of Caco2 cells through Matrigel (Figure 1H), which could be blocked by the HPSE inhibitor SST0001 (Roneparstat), a glycol-split heparin (40) (Figure 1H), suggesting a mechanistic role for HPSE upregulation in Sdc-1 deficient cells in this process. Sdc-1 depletion resulted in a downregulation of the epithelial cell adhesion molecule E-cadherin and an upregulation of the mesenchymal marker vimentin (Figure 1I), suggesting a possible involvement of EMT in this process. Notably, the Sdc-1-dependent upregulation of vimentin could be abolished by the HPSE inhibitor SST0001, consistent with its inhibitory effect in the invasion assay (Figures 1H,I). To analyze the interdependence of Sdc-1 and HPSE expression, we tested the impact of Sdc-1 depletion on expression of the transcription factor Egr1, a known regulator of HPSE expression (21, 41). qPCR and Western blot analysis revealed an upregulation of Egr1 in Sdc-1-depleted cells (Figures 2A,B). Notably, siRNA depletion of EGR1 abolished the upregulation of HPSE mRNA expression (Figure 2C) and dampened HPSE promoter activation (Figure 2D) in Sdc-1-depleted cells. Notably, the increased activity of FAK in Sdc-1-depleted cells was abolished by EGR1 siRNA knockdown (Figure 2E), whereas application of a FAK inhibitor resulted in an inhibition of Sdc-1-dependent EGR1 and HPSE expression (Figures 2F,G), indicating a mechanistic involvement of this pathway.

### Heparanase Regulates the Cancer Stem Cell Phenotype of Caco2 Cells

**Figure 3:**
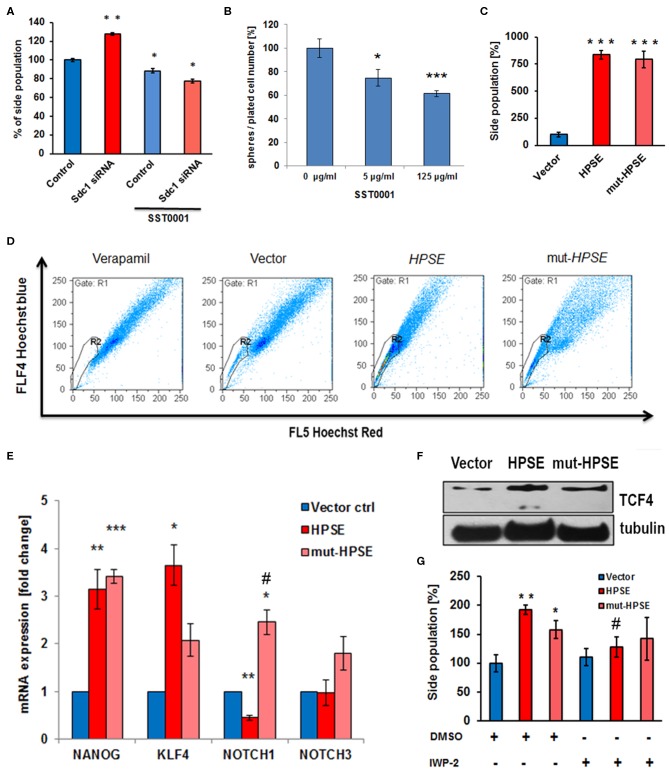
Heparanase regulates the cancer stem cell phenotype of Caco2 cells. **(A)** Sdc-1 siRNA knockdown and heparanase inhibition by SST0001 affect the stem cell marker side population in opposite directions. **p < 0.01 vs. all groups. *p < 0.05 vs. untreated control. **(B)** The HPSE inhibitor SST0001 (10 μg/ml) reduces sphere formation as a readout of stem cell acivity. ***p < 0.001, *p < 0.05 vs. untreated control. **(C,D)** Overexpression of native and enzymatically inactive forms of HPSE markedly increases the Caco2 side population. ***p < 0.001 vs. vector control. **(C)** Quantification of flow cytometric data. **(D)** representative flow cytometric measurements. Verapamil = inhibitor control. **(E,F)** Overexpression of native and enzymatically inactive forms of HPSE differentially affect the expression of the stem cell markers NANOG, KLF4, NOTCH1, NOTCH3, and TCF4. **(E)** qPCR, ***p < 0.001, **p < 0.01, *p < 0.05 vs. vector control, #*p* < 0.05 vs. HPSE. **(F)** Western-Blot. **(G)** The Wnt pathway inhibitor IWP2 reduces the enhancing effect of HPSE overexpression on the side population phenotype. **p < 0.01, *p < 0.05 vs. control, #*p* < 0.05 vs. untreated HPSE. All panels *N* ≥ 3. Error bars = SEM. **(D,F)** representative example of three independent experiments.

**Figure 4:**
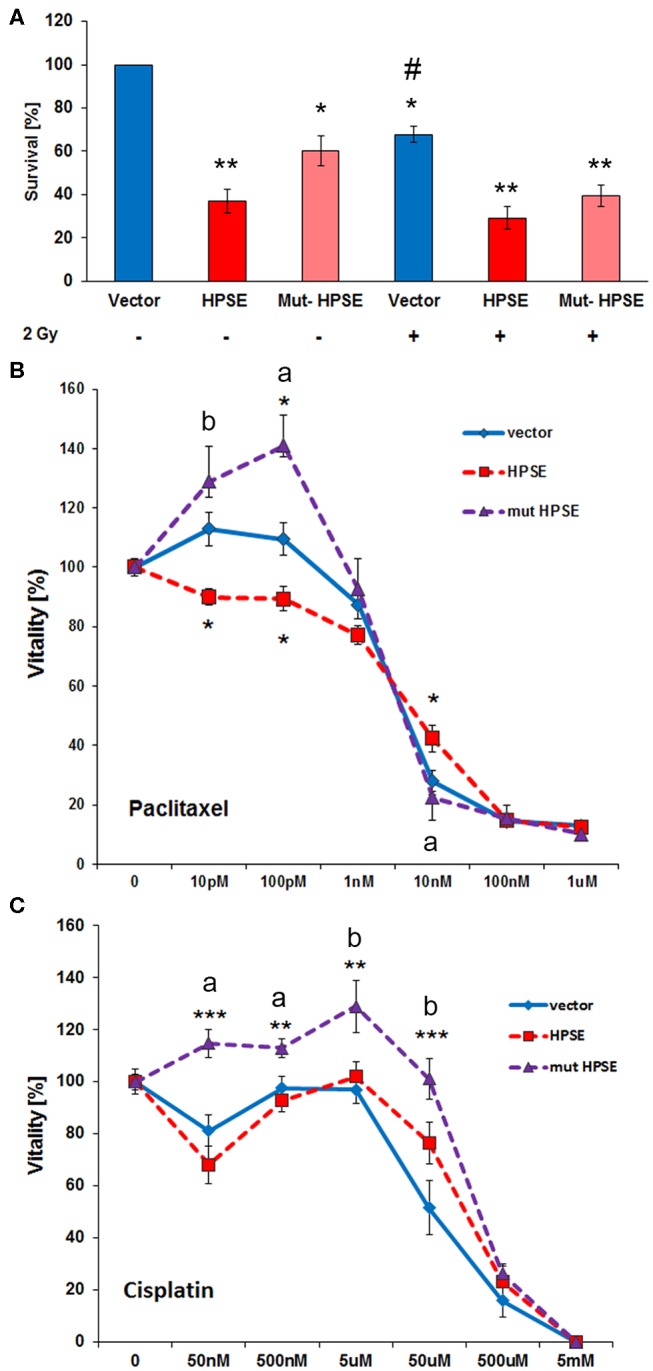
Enzymatically active and inactive forms of heparanase differentially affect the resistance of Caco2 cells to chemotherapeutics and radiation treatment. **(A)** Vector control and HPSE **/** mutant HPSE overexpressing Caco2 cells were subjected to irradiation with 2 Gy and then to a colony formation assay as a readout of cell survival. Compared to controls, HPSE overexpressing cells showed reduced colony formation. Only control cells showed a significant radiation-induced reduction in colony formation. **(B)** Overexpression of enzymatically inactive HPSE increases Caco2 resistance to Paclitaxel chemotherapy, whereas HPSE reduces resistance at low treatment doses. MTT cell viability assay. **(C)** Overexpression of enzymatically inactive HPSE increases Caco2 resistance to Cisplatin chemotherapy. MTT cell viability assay. All panels *N* ≥ 3. Error bars = SEM, ***p < 0.001, **p < 0.01, *p < 0.05 vs. control. ^a^*p* < 0.01 vs. HPSE, ^*b*^*p* < 0.05 vs. HPSE. #*p* < 0.05 vs. unirradiated control within the same treatment group.

Altered Sdc-1 expression has been linked to aberrant CSC function, a phenotype linked to therapeutic resistance and cancer recurrence (3, 47). To test a possible involvement of the Sdc-1-HPSE axis in this phenotype, we analyzed several readouts of stem cell activity in our cells. Sdc-1 knockdown enhanced the CSC-associated side population (SP) phenotype (Figure 3A). Notably, the HPSE inhibitor SST0001 abolished this effect (Figure 3A) and inhibited the formation of colonospheres in wild-type cells (Figure 3B). While upregulation of both enzymatically active and inactive HPSE forms massively increased the SP phenotype (Figures 3C,D), upregulation of the stemness-associated transcription factors Krüppel-like factor 4 (KLF4) and transcription factor 4 (TCF4) was more pronounced in cells overexpressing native HPSE (Figures 3E,F). Expression of NANOG was upregulated by both forms of HPSE, whereas NOTCH1 expression was differentially affected by the catalytically active and inactive forms of HPSE (Figure 3E). Application of the Wnt-pathway inhibitor IWP2 reduced the effect of HPSE expression on the side population phenotype (Figure 3G). Overall, these data suggest that HPSE regulates CSC properties by affecting multiple stemness-associated signaling pathways. As CSC function has been linked to therapeutic resistance (3), we finally tested the influence of HPSE overexpression on resistance to irradiation and chemotherapy. The Caco2 colony formation capacity under control conditions was reduced by HPSE overexpression compared to vector controls. However, upon radiation with a therapeutically relevant dose of 2 Gy, HPSE overexpressing cells showed no significant decrease in colony formation capacity, whereas colony formation was significantly decreased in control cells (Figure 4A). Chemosensitivity assays revealed an increased resistance of cells overexpressing enzymatically inactive HPSE to paclitaxel and cisplatin, whereas upregulation of enzymatically active HPSE has either no effect (cisplatin) or a mixed, dose-dependent effect (paclitaxel) (Figures 4B,C). Taken together, our data suggest that HPSE overexpression is associated with changes in the resistance of colon cancer cells to chemo- and radio- therapy, involving a differential role for the enzymatic activity of HPSE.

## Discussion

The cell surface proteoglycan Sdc-1 acts as an ECM adhesion receptor and co-receptor for numerous signaling pathways with relevance to tumor progression (23, 48). The Sdc-1 heparan sulfate chains serve as substrates for HPSE, and this degradative process modulates tumor angiogenesis, growth factor-dependent tumor cell proliferation and metastatic behavior in a variety of tumor entities (8). Notably, a decrease in Sdc-1 and increase in HPSE expression has been observed in several cancers particularly in colon cancer enhancing tumorigenesis, invasion, and metastasis (7–15). The molecular mechanism underlying this regulation has not been resolved. Our results demonstrate that (i) loss of Sdc-1 enhances transcriptional regulation of HPSE and vice versa, (ii) increased Sdc-1-dependent HPSE expression increases invasiveness and can be reversed by HPSE inhibition, (iii) molecular cross-talk between EGR1 and activation of FAK upon loss of Sdc-1 collectively drive HPSE expression, (iv) this expression boosts colon CSC properties, and (v) these processes are associated with alterations in the resistance of Caco2 cells to radio- and chemotherapy *in vitro*.

Increased expression of EGR1, an early growth response gene mediated by Sdc-1 downregulation, correlated with the increase in HPSE expression. Mutagenesis and trans-activation studies have previously shown that EGR1 binds to the HPSE promoter and up-regulates HPSE transcription in colon cancer cells (21). Our results further support that EGR1 directly regulates HPSE transcription. Data in fibrosarcoma cells suggest that the nuclear localization of Sdc-1 is a critical factor in regulating EGR1 expression, as expression of Sdc-1 lacking its nuclear localization signal resulted in upregulation of EGR1 (49). Therefore, the upregulation of EGR1 observed in Sdc-1-depleted Caco2 cells in our study may have been due to the reduction in nuclear, Sdc-1. Increased invasion of Sdc-1-depleted cells may be due to the degradation of HS chains, which impairs cell-cell contact and cell-matrix adhesion interactions. HPSE controls cell barrier function attributed to its HS degradative and Sdc-1 sheddase activity (8), suggesting that cleavage of Sdc-1 at the cell membrane will favor proinvasive conditions. Several pharmacodynamic studies have demonstrated that the HPSE inhibitor SST0001, has anti-tumor activity in different cancer models (40). Given that HPSE has multiple functions in the tumor microenvironment it is conceivable that SST0001 decreases the invasion of Sdc-1 depleted cells through HPSE-mediated signaling events (50, 51), or via direct inhibition of its basement-membrane degrading properties. Due to the loss of heparan sulfate, epithelial cells lose their cell polarity and gain migratory and invasive properties via the process of epithelial to mesenchymal transition (EMT). Indeed, in our experimental system, Sdc-1-depletion resulted in a downreguation of the antimetastatic epithelial cell adhesion molecule E-cadherin, and an upregulation of the mesenchymal marker vimentin. Although the underlying molecular processes remain poorly understood, it was reported that Sdc-1 depletion enhances formation of lamellipodia associated with an increase in invasive capabilities (42). It is well-documented that HSPG bind several EMT-inducing factors, such as FGF, hepatocyte growth factor and transforming growth factor-β. Therefore, enhanced HPSE expression may liberate these bound factors and thereby further enhancing EMT-like conditions (52). Moreover, we could show that HPSE inhibition could revert the upregulation of mesenchymal vimentin observed in Sdc-1-depleted cells. Also, a shift of Sdc-1 from epithelial to stromal cells might attenuate the antimetastatic effect of Sdc-1 at the cancer cell surface where loss of its expression can promote EMT (53).

A mountain of evidence shows that EMT-like conditions promote proliferation, metastasis, chemo-, immune- and radiotherapy resistance, all of which are relevant to cancer stem cell properties (26–28, 54). In breast cancer, enhanced activation of integrins caused by Sdc-1 downregulation results in increased FAK activation (33). We, therefore, hypothesized that FAK might be involved in HPSE regulation. Indeed, blocking FAK autophosphorylation decreased HPSE expression in the absence of Sdc-1 (Figure 2G). It was previously reported that a putative HPSE receptor activates the phosphoinositide 3-kinase-protein kinase B (AKT) pathway (51), but it is not clear whether phosphorylated FAK activates this receptor. However, PF562271 effectively abolished heparanase-induced AKT activation (51), consistent with our results, where FAK inhibitor attenuated HPSE expression. It was reported that integrin/epidermal growth factor receptor cross-talk dependent adhesion signals regulate EGR1 expression (55). It is conceivable that upon Sdc-1 loss, the beta1 integrin complex on the plasma membrane may trigger the expression of EGR1 through adhesion-dependent signals, which would further lead to the activation of FAK. Overall, these data demonstrate that upon Sdc-1 loss, an EGR1/pFAK cross-talk is required for expression of HPSE through a novel regulatory signaling cascade, opening new strategies for therapeutic intervention.

Previous data from our group have indicated a role for Sdc-1 in CSC function (3, 31, 47), including an impact on the side population. Here, we demonstrate that basal sphere formation of our Caco2 model cell line and the Sdc-1 knockdown-induced increase in the SP can be blocked by HPSE inhibition, whereas upregulation of HPSE results in a substantial increase of this surrogate stem cell marker, independent of HPSE enzymatic activity. These results ascribe, for the first time, a role for HPSE in regulating CSC properties, and an impact of the HPSE inhibitor SST0001 on SP levels. As SST0001 profoundly decreased invasion of Sdc-1 depleted cells, it is conceivable that genes involved in cell invasion may also further regulate SP and/or that SST0001 is directly acting on genes associated with stemness. The significant increase in the SP as a result of HPSE overexpression provides further evidence for the multifunctional roles of HPSE in the tumor microenvironment. At the mRNA level, we saw a high increase in the expression of NANOG and KLF4 in cells expressing either native or mutant HPSE. Indeed there are indications for cell adhesion-dependent functions of enzymatically inactive HPSE (56, 57). The increase in NOTCH1 and NOTCH3 in mutant HPSE expressing cells could explain the increase in the SP seen in dominant negative clones (47, 58). During progression of the primary tumor, HPSE, by promoting autocrine and paracrine signaling functions, appears to initiate non-stem cell epithelial cells to develop into tumor-initiating cells via the re-expression of stem cell markers, including pluripotency-associated transcription factors (9). A range of signals were shown to regulate the tumor-initiating stem cell capacities of colon cancer, including the Wnt pathway (59). We observed a high expression of TCF4 in HPSE-overexpressing clones. Our results furthermore showed a decrease in the SP upon incubation with IWAP2 that inhibits the palmitylation of Wnt proteins and thereby blocks Wnt secretion and activity (60).

CSCs have been implicated in resistance to irradiation and chemotherapy due to increased expression of MDR proteins and highly efficient DNA repair mechanisms (3, 7). Irradiated HPSE overexpressing cells showed partial radioresistance compared to untreated controls. In addition, we observed increased chemoresistance in cells expressing mutant HPSE. The mutant inactive form of heparanase is involved in adhesion-dependent signaling which in turn may promote chemoresistance of cancer cells by increasing the side population (8, 57). It is also important to consider that the SP is controlled by several additional factors including, genetic alterations, the ECM niche microenvironment, micro RNA's, stem cells and their quiescent vs. active state (61). Altogether, the nature of drug resistance of tumor-initiating cells is multifactorial, with various signaling pathways and complex mechanisms that could fine-tune chemosensitivity.

To summarize, we have shown for the first time the involvement of HPSE in colon cancer stem cell properties and observed an increase in cell invasiveness linked to the regulatory interplay of Sdc-1 and HPSE. Moreover, we identified relevant signaling pathways (FAK, Wnt, Notch) and transcription factors (Egr1, TCF4), as constituents of this regulatory circuit, which paves the way for a more efficient combinatorial targeting of colon cancer in the context of therapeutic resistance.

## Data Availability Statement

All datasets generated for this study are included in the article/supplementary material.

## Author Contributions

SK performed the majority of experiments, analyzed data, drafted figures, and the initial version of the manuscript. PP performed molecular biology and stem cell culture experiments. CC performed stem cell culture experiments. RR supervised and performed molecular biology and stem cell culture experiments, analyzed data, and designed experiments. IV provided essential reagents and expertise on HPSE, revised the manuscript. BG assisted with, and analyzed flow cytometric and irradiation experiments, designed experiments, cosupervised the study, drafted figures. MG conceived and supervised the study, analyzed data drafted the final figures, and wrote the manuscript. All authors have read, revised, and approved the final version of the manuscript.

## Conflict of Interest

The authors declare that the research was conducted in the absence of any commercial or financial relationships that could be construed as a potential conflict of interest.

## References

[1] Barker HE Paget JT Khan AA Harrington KJ. The tumour microenvironment after radiotherapy: mechanisms of resistance and recurrence. Nat Rev Cancer. (2015) 15:409–25. 10.1038/nrc3958 26105538 PMC4896389

[2] Gialeli C Theocharis AD Karamanos NK. Roles of matrix metalloproteinases in cancer progression and their pharmacological targeting. FEBS J. (2011) 278:16–27. 10.1111/j.1742-4658.2010.07919.x 21087457

[3] Vitale D Kumar Katakam S Greve B Jang B Oh ES Alaniz L. Proteoglycans and glycosaminoglycans as regulators of cancer stem cell function and therapeutic resistance. FEBS J. (2019) 286:2870–82. 10.1111/febs.14967 31230410

[4] Filatova A Acker T Garvalov BK. The cancer stem cell niche(s): the crosstalk between glioma stem cells and their microenvironment. Biochim Biophys Acta. (2013) 1830:2496–508. 10.1016/j.bbagen.2012.10.008 23079585

[5] Ibrahim SA Hassan H Götte M. MicroRNA regulation of proteoglycan function in cancer. FEBS J. (2014) 281:5009–22. 10.1111/febs.13026 25168226

[6] Vijaya Kumar A Salem Gassar E Spillmann D Stock C Sen YP Zhang T. HS3ST2 modulates breast cancer cell invasiveness via MAP kinase-and Tcf4(Tcf7l2)- dependent regulation of protease and cadherin expression. Int J Cancer. (2014) 135:2579–92. 10.1002/ijc.28921 24752740

[7] Götte M Yip GW. Heparanase, hyaluronan, and CD44 in cancers: a breast carcinoma perspective. Cancer Res. (2006) 66:10233–7. 10.1158/0008-5472.CAN-06-1464 17079438

[8] Vlodavs ky I Singh P Boyango I Gutter-Kapon L Elkin M Sanderson RD Heparanase: from basic research to therapeutic applications in cancer and inflammation. Drug Resist Updat. (2016) 29:54–75. 10.1016/j.drup.2016.10.001 27912844 PMC5447241

[9] Friedmann Y Vlodavs.ky I Aingorn H Aviv A Peretz T Pecker I. Expression of heparanase in normal, dysplastic, and neoplastic human colonic mucosa and stroma. Evidence for its role in colonic tumorigenesis. Am J Pathol. (2000) 157:1167–75. 10.1016/S0002-9440(10)64632-9 11021821 PMC1850180

[10] Hashimoto Y Skacel M Adams JC. Association of loss of epithelial syndecan-1 with stage and local metastasis of colorectal adenocarcinomas: an immunohistochemical study of clinically annotated tumors. BMC Cancer. (2008) 8:185. 10.1186/1471-2407-8-185 18590537 PMC2459187

[11] Fernández-Vega I García-Suárez O García B Crespo A Astudillo A Quirós LM. Heparan sulfate proteoglycans undergo differential expression alterations in right sided colorectal cancer, depending on their metastatic character. BMC Cancer. (2015) 15:742. 10.1186/s12885-015-1724-9 26482785 PMC4617710

[12] Crespo A García-Suárez O Fernández-Vega I Solis-Hernandez MP García B Castañón S. Heparan sulfate proteoglycans undergo differential expression alterations in left sided colorectal cancer, depending on their metastatic character. BMC Cancer. (2018) 18:687. 10.1186/s12885-018-4597-x 29940912 PMC6019305

[13] Fujiya M Watari J Ashida T Honda M Tanabe H Fujiki T. Reduced expression of syndecan-1 affects metastatic potential and clinical outcome in patients with colorectal cancer. Jpn J Cancer Res. (2001) 92:1074–81. 10.1111/j.1349-7006.2001.tb01062.x 11676858 PMC5926619

[14] Takaoka M Naomoto Y Ohkawa T Uetsuka H Shirakawa Y Uno F. Heparanase expression correlates with invasion and poor prognosis in gastric cancers. Lab Invest. (2003) 83:613–22. 10.1097/01.LAB.0000067482.84946.BD 12746471

[15] Levy P Munier A Baron-Delage S Di Gioia Y Gespach C Capeau J Syndecan-1 alterations during the tumorigenic progression of human colonic Caco-2 cells induced by human Ha-ras or polyoma middle T oncogenes. Br J Cancer. (1996) 74:423–31. 10.1038/bjc.1996.376 8695359 PMC2074646

[16] Lundin M Nordling S Lundin J Isola J Wiksten JP Haglund C. Epithelial syndecan-1 expression is associated with stage and grade in colorectal cancer. Oncology. (2005) 68:306–13. 10.1159/000086969 16020957

[17] Lerner I Hermano E Zcharia E Rodkin D Bulvik R Doviner V. Heparanase powers a chronic inflammatory circuit that promotes colitis-associated tumorigenesis in mice. J Clin Invest. (2011) 121:1709–21. 10.1172/JCI43792 21490396 PMC3083784

[18] Doviner V Maly B Kaplan V Gingis-Velitski S Ilan N Vlodavs.ky I. Spatial and temporal heparanase expression in colon mucosa throughout the adenoma-carcinoma sequence. Mod Pathol. (2006) 19:878–88. 10.1038/modpathol.3800603 16607375

[19] Nobuhisa T Naomoto Y Ohkawa T Takaoka M Ono R Murata T. Heparanase expression correlates with malignant potential in human colon cancer. J Cancer Res Clin Oncol. (2005) 131:229–37. 10.1007/s00432-004-0644-x 15625607 PMC12161232

[20] Mikami S Ohashi K Usui Y Nemoto T Katsube K Yanagishita M. Loss of syndecan-1 and increased expression of heparanase in invasive esophageal carcinomas. Jpn J Cancer Res. (2001) 92:1062–73. 10.1111/j.1349-7006.2001.tb01061.x 11676857 PMC5926620

[21] de Mestre AM Rao S Hornby JR Soe-Htwe T Khachigian LM Hulett MD. Early growth response gene 1 (EGR1) regulates heparanase gene transcription in tumor cells. J Biol Chem. (2005) 280:35136–47. 10.1074/jbc.M503414200 16093249

[22] Ilan N Elkin M Vlodavs.ky I. Regulation, function and clinical significance of heparanase in cancer metastasis and angiogenesis. Int J Biochem Cell Biol. (2006) 38:2018–39. 10.1016/j.biocel.2006.06.004 16901744

[23] Götte M Kovalszky I. Extracellular matrix functions in lung cancer. Matrix Biol. (2018) 73:105–21. 10.1016/j.matbio.2018.02.018 29499357

[24] Visvader JE Lindeman GJ. Cancer stem cells in solid tumours: accumulating evidence and unresolved questions. Nat Rev Cancer. (2008) 8:755–68. 10.1038/nrc2499 18784658

[25] Brabletz T Jung A Spaderna S Hlubek F Kirchner T. Opinion: migrating cancer stem cells - an integrated concept of malignant tumour progression. Nat Rev Cancer. (2005) 5:744–9. 10.1038/nrc1694 16148886

[26] Mani SA Guo W Liao MJ Eaton EN Ayyanan A Zhou AY. The epithelial-mesenchymal transition generates cells with properties of stem cells. Cell. (2008) 133:704–15. 10.1016/j.cell.2008.03.027 18485877 PMC2728032

[27] Aktas B Tewes M Fehm T Hauch S Kimmig R Kasimir-Bauer S. Stem cell and epithelial-mesenchymal transition markers are frequently overexpressed in circulating tumor cells of metastatic breast cancer patients. Breast Cancer Res. (2009) 11:R46. 10.1186/bcr2333 19589136 PMC2750105

[28] Piperigkou Z Franchi M Riethmüller C Götte M Karamanos NK miR-200b restrains EMT and aggressiveness and regulates matrix composition depending on ER status and signaling in mammary cancer. Matrix Biol Plus. (in press). 10.1016/j.mbplus.2020.100024 PMC7852204 33543022

[29] Mahtouk K Hose D Raynaud P Hundemer M Jourdan M Jourdan E. Heparanase influences expression and shedding of syndecan-1, and its expression by the bone marrow environment is a bad prognostic factor in multiple myeloma. Blood. (2007) 109:4914–23. 10.1182/blood-2006-08-043232 17339423 PMC2268882

[30] Ramani VC Purushothaman A Stewart MD Thompson CA Vlodavs.ky I Au JL. The heparanase/syndecan-1 axis in cancer: mechanisms and therapies. FEBS J. (2013) 280:2294–306. 10.1111/febs.12168 23374281 PMC3651779

[31] Ibrahim SA Hassan H Vilardo L Kumar SK Kumar AV Kelsch R. Syndecan-1 (CD138) modulates triple-negative breast cancer stem cell properties via regulation of LRP-6 and IL-6-mediated STAT3 signaling. PLoS ONE. (2013) 8:e85737. 10.1371/journal.pone.0085737 24392029 PMC3877388

[32] Ridgway LD Wetzel MD Marchetti D. Heparanase modulates Shh and Wnt3a signaling in human medulloblastoma cells. Exp Ther Med. (2011) 2:229–38. 10.3892/etm.2010.189 21442027 PMC3063606

[33] Hassan H Greve B Pavao MS Kiesel L Ibrahim SA Götte M. Syndecan-1 modulates β-integrin-dependent and interleukin-6-dependent functions in breast cancer cell adhesion, migration, and resistance to irradiation. FEBS J. (2013) 280:2216–27. 10.1111/febs.12111 23289672

[34] Spyrou A Kundu S Haseeb L Yu D Olofsson T Dredge K. Inhibition of heparanase in pediatric brain tumor cells attenuates their proliferation, invasive capacity, and *in vivo* tumor growth. Mol Cancer Ther. (2017) 16:1705–16. 10.1158/1535-7163.MCT-16-0900 28716813

[35] Xing Y Cui D Wang S Wang P Xing X Li H. Oleuropein represses the radiation resistance of ovarian cancer by inhibiting hypoxia and microRNA-299-targetted heparanase expression. Food Funct. (2017) 8:2857–64. 10.1039/C7FO00552K 28726915

[36] Davidson B Shafat I Risberg B Ilan N Trope' CG Vlodavs.ky I. Heparanase expression correlates with poor survival in metastatic ovarian carcinoma. Gynecol Oncol. (2007) 104:311–9. 10.1016/j.ygyno.2006.08.045 17030350

[37] Shteingauz A Boyango I Naroditsky I Hammond E Gruber M Doweck I. Heparanase enhances tumor growth and chemoresistance by promoting autophagy. Cancer Res. (2015) 75:3946–57. 10.1158/0008-5472.CAN-15-0037 26249176 PMC4573896

[38] Wang Z Götte M Bernfield M Reizes O. Constitutive and accelerated shedding of murine syndecan-1 is mediated by cleavage of its core protein at a specific juxtamembrane site. Biochemistry. (2005) 44:12355–61. 10.1021/bi050620i 16156648 PMC2546870

[39] Hulett MD Hornby JR Ohms SJ Zuegg J Freeman C Gready JE. Identification of active-site residues of the pro-metastatic endoglycosidase heparanase. Biochemistry. (2000) 39:15659–67. 10.1021/bi002080p 11123890

[40] Lanzi C Zaffaroni N Cassinelli G. Targeting heparan sulfate proteoglycans and their modifying enzymes to enhance anticancer chemotherapy efficacy and overcome drug resistance. Curr Med Chem. (2017) 24:2860–86. 10.2174/0929867324666170216114248 28215163

[41] Meirovitz A Hermano E Lerner I Zcharia E Pisano C Peretz T. Role of heparanase in radiation-enhanced invasiveness of pancreatic carcinoma. Cancer Res. (2011) 71:2772–80. 10.1158/0008-5472.CAN-10-3402 21447736 PMC3070855

[42] Ibrahim SA Yip GW Stock C Pan JW Neubauer C Poeter M. Targeting of syndecan-1 by microRNA miR-10b promotes breast cancer cell motility and invasiveness via a Rho-GTPase- and E-cadherin-dependent mechanism. Int J Cancer. (2012) 131:E884–96. 10.1002/ijc.27629 22573479

[43] Götte M Greve B Kelsch R Müller-Uthoff H Weiss K Kharabi Masouleh B The adult stem cell marker musashi-1 modulates endometrial carcinoma cell cycle progression and apoptosis via notch-1 and p21(WAF1/CIP1). Int J Cancer. (2011) 129:2042–49. 10.1002/ijc.25856 21165952

[44] Greve B Kelsch R Spaniol K Eich HT Götte M. Flow cytometry in cancer stem cell analysis and separation. Cytometry A. (2012) 81:284–93. 10.1002/cyto.a.22022 22311742

[45] Elkin M Cohen I Zcharia E Orgel A Guatta-Rangini Z Peretz T. Regulation of heparanase gene expression by estrogen in breast cancer. Cancer Res. (2003) 63:8821–6. 14695198

[46] Zcharia E Zilka R Yaar A Yacoby-Zeevi O Zetser A Metzger S. Heparanase accelerates wound angiogenesis and wound healing in mouse and rat models. FASEB J. (2005) 19:211–21. 10.1096/fj.04-1970com 15677344

[47] Ibrahim SA Gadalla R El-Ghonaimy EA Samir O Mohamed HT Hassan H. Syndecan-1 is a novel molecular marker for triple negative inflammatory breast cancer and modulates the cancer stem cell phenotype via the IL-6/STAT3, notch and EGFR signaling pathways. Mol Cancer. (2017) 16:57. 10.1186/s12943-017-0621-z 28270211 PMC5341174

[48] Espinoza-Sánchez NA Götte M. Role of cell surface proteoglycans in cancer immunotherapy. Semin Cancer Biol. (2019) 62:48–67. 10.1016/j.semcancer.2019.07.012 31336150

[49] Szatmári T Mundt F Kumar-Singh A Möbus L Ötvös R Hjerpe A Dobra K. Molecular targets and signaling pathways regulated by nuclear translocation of syndecan-1. BMC Cell Biol. (2017) 18:34. 10.1186/s12860-017-0150-z 29216821 PMC5721467

[50] Cassinelli G Lanzi C Tortoreto M Cominetti D Petrangolini G Favini E. Antitumor efficacy of the heparanase inhibitor SST0001 alone and in combination with antiangiogenic agents in the treatment of human pediatric sarcoma models. Biochem Pharmacol. (2013) 85:1424–32. 10.1016/j.bcp.2013.02.023 23466421

[51] Riaz A Ilan N Vlodavs.ky I Li JP Johansson S. Characterization of heparanase-induced phosphatidylinositol 3-kinase-AKT activation and its integrin dependence. J Biol Chem. (2013) 288:12366–75. 10.1074/jbc.M112.435172 23504323 PMC3636920

[52] Kirkbride KC Ray BN Blobe GC. Cell-surface co-receptors: emerging roles in signaling and human disease. Trends Biochem Sci. (2005) 30:611–21. 10.1016/j.tibs.2005.09.003 16185874

[53] Mennerich D Vogel A Klaman I Dahl E Lichtner RB Rosenthal A. Shift of syndecan-1 expression from epithelial to stromal cells during progression of solid tumours. Eur J Cancer. (2004) 40:1373–82. 10.1016/j.ejca.2004.01.038 15177497

[54] Nieto MA Cano A. The epithelial-mesenchymal transition under control: global programs to regulate epithelial plasticity. Semin Cancer Biol. (2012) 22:361–8. 10.1016/j.semcancer.2012.05.003 22613485

[55] Cabodi S Morello V Masi A Cicchi R Broggio C Distefano P. Convergence of integrins and EGF receptor signaling via PI3K/Akt/FoxO pathway in early gene Egr-1 expression. J Cell Physiol. (2009) 218:294–303. 10.1002/jcp.21603 18844239

[56] Goldshmidt O Zcharia E Cohen M Aingorn H Cohen I Nadav L. Heparanase mediates cell adhesion independent of its enzymatic activity. FASEB J. (2003) 17:1015–25. 10.1096/fj.02-0773com 12773484

[57] Levy-Adam F Ilan N Vlodavs.ky I. Tumorigenic and adhesive properties of heparanase. Semin Cancer Biol. (2010) 20:153–60. 10.1016/j.semcancer.2010.06.005 20619346 PMC2941534

[58] Shibue T Brooks MW Weinberg RA. An integrin-linked machinery of cytoskeletal regulation that enables experimental tumor initiation and metastatic colonization. Cancer Cell. (2013) 24:481–98. 10.1016/j.ccr.2013.08.012 24035453 PMC3864118

[59] Yamada T Takaoka AS Naishiro Y Hayashi R Maruyama K Maesawa C. Transactivation of the multidrug resistance 1 gene by T-cell factor 4/beta-catenin complex in early colorectal carcinogenesis. Cancer Res. (2000) 60:4761–6. 10987283

[60] Chikazawa N Tanaka H Tasaka T Nakamura M Tanaka M Onishi H. Inhibition of Wnt signaling pathway decreases chemotherapy-resistant side-population colon cancer cells. Anticancer Res. (2010) 30:2041–8. 20651349

[61] Richard V Nair MG Santhosh Kumar TR Pillai MR. Side population cells as prototype of chemoresistant, tumor-initiating cells. Biomed Res Int. (2013) 2013:517237. 10.1155/2013/517237 24294611 PMC3834974

[62] Kumar Katakam S Syndecan-1 and heparanase as novel regulators of colon cancer stem cell function (Ph.D. thesis). Faculty of Biology, Westfälische-Wilhelms-Universität Münster, Münster, Germany (2015).

